# The impact of oral health on quality of life of urban and riverine populations of the Amazon: A multilevel analysis

**DOI:** 10.1371/journal.pone.0208096

**Published:** 2018-11-30

**Authors:** Camila de Vasconcellos Rocha Maia, Fausto Medeiros Mendes, David Normando

**Affiliations:** 1 Dental School, Institute of Health Sciences, Federal University of Pará, Belém, Pará, Brazil; 2 Dental School, Department of Orthodontics and Pediatric Dentistry, State University of São Paulo, São Paulo, Brazil; 3 Dental School, Department of Orthodontics, Federal University of Pará, Belém, Pará, Brazil; Griffith University, AUSTRALIA

## Abstract

Few studies and epidemiological surveys are carried out in populations geographically and culturally isolated, such as rural and riverine communities. Riverine populations represent one of the typical populations of the Amazon region. This study aimed to evaluate the impact of dental caries and periodontal disease on the quality of life of adolescents and young adults from an urban area and from two riverine communities of the Amazon region located at different distances from the urban area. The occurrence of dental caries and periodontal disease was examined through the Decayed, Missing, Filled (DMFT) Index and the Community Periodontal Index (CPI). The impact of oral health conditions on quality of life was examined through the Oral Health Impact Profile (OHIP-14) questionnaire. The data were collected from 564 individuals (15–25 years old): 212 residents of the urban area of Abaetetuba-Pará-Brazil, were compared to 186 inhabitants of the nearest riverine community (Maracapucu) and 166 of another riverine community (Tucumanduba River) located further from the city. The OHIP-14 was analyzed as a dependent outcome, while dental caries, bleeding, calculus, the presence of periodontal pockets, as well as sex and age were analyzed as independent variables through Poisson analysis in a multilevel model. The worst indicators of OHIP-14 and the highest DMFT scores were found in the riverine populations, especially those from the riverine community farthest from the urban area. Based on the adjusted multivariate model, variables such as the contextual variable (location, RR 1.31), and individual demographic variables (sex and age), DMFT (RR 1.53), and the presence of periodontal pockets (RR 1.15) were significantly related to the OHIP (p<0.001). Our results confirm that dental caries and periodontal disease negatively impact oral health-related quality of life; however, these diseases seem to impact the individuals from remote communities more significantly.

## Introduction

Riverine peoples are typical populations of the Amazon region. They inhabit the river borders and depend on the river for food, transportation, work, and subsistence [1, 2]. Daily life is essentially associated with the seasonality of the rivers, and local economy is based on arbitrary fishing and small scale farming. Epidemiological studies in these communities are scarce due to the difficulties of access and logistics, inefficient transportation systems, insufficient work conditions for researchers, demographic dispersion of the population along the rivers, and the absence of socio-demographic records.

Different from what can be observed regarding rural/riverine populations, several studies are conducted in urban populations by means of epidemiological data in oral health. These studies have proved that high frequency oral diseases- such as dental caries and periodontal disease- influence the quality of life [3–6]. This relationship is determined by the health condition of adjacent teeth and buccal tissues, which allows the individual to feed and chew without difficulty and to obtain aesthetic satisfaction and absence of pain [7–9].

Identifying factors that affect the quality of life of riverine communities is extremely relevant. These factors may provide insights for other remote communities both in Brazil and around the globe that share the same characteristics of social, cultural, and economical isolation or semi-isolation from more urban communities and/or are organized around a different sociocultural pattern.

Studies have shown that oral health is worse at lower levels of household income [10] and that at a country level, the utilization of dental care is inversely associated with income inequality [11]. Due to the economic and social deprivation in which these populations live, their oral health conditions may result in higher vulnerability in comparison to urban populations.

On the other hand, communities’ isolation and consequent lack of urban commercial behavior may lead to healthier eating, rich in fish and organic products with lower consumption of industrialized products, ultimately contributing to better oral health conditions. Thus, considering cultural peculiarities, as well as constraints in information access and the subjective recognition of the health–disease process, it is possible to recognize differences among urban and remote populations in relation to the impact of oral health on the quality of life.

From this perspective, this article aims to evaluate the impact of dental caries and periodontal disease on riverine populations’ quality of life. In order to achieve this objective, the study includes a comparative analysis between adolescents and young adults of one urban and two riverine communities in the Amazon region.

## Materials and methods

The Research Ethics Committee of the Health Sciences Institute of the Federal University of Pará approved the development of this study under protocol no. 1,593,113, dated June 2016, S1 File. The data acquisition occurred from September 2016 to April 2017.

### Study design, setting, and ethical considerations

This study has a cross-sectional design and conforms to STROBE guidelines [12]. Its study group is urban and riverine public schools in the municipality of Abaetetuba (State of Pará, Amazon, Brazil), which is the seventh most populated in the state and is marked by a vast hydrographic landscape, navigable in almost all its extension. Abaetetuba also has the largest number of minor islands in the state—about 45 islands that form the so-called Islands Region—inhabited by several riverine communities that live semi-isolated, with no basic sanitation services and difficult access to the urban center. The local estimated population is 153,380 inhabitants (2017): 90,830 inhabitants in the main town area and 62,550 inhabitants in surrounding country areas. Among the rural population, approximately 37,000 inhabitants are riverine. The municipality does not have fluoridated water, and the Human Development Index (HDI) was 0.628 in 2010 [13].

### Participants and study extent

The study sample includes 564 adolescents and young adults from 15 to 25 years old, enrolled in one urban (*n* = 212) and two riverine local public schools: Maracapucu community (*n* = 186) and Tucumanduba community (*n* = 166). The upper age group was considered due to longer teeth exposure to the oral environment, and the frequent occurrence of this age range in remote school environments. The school selection was based on convenience for the urban area. The school is located downtown Abaetetuba, while the riverine schools were selected based on the presence of secondary education to reach students within the pre-established age range.

We examined and surveyed individuals that met de age criterium and were present at their respective school period—morning, afternoon, or evening—except for those who refused to participate in the study. A free informed consent form (ICF) signed by individuals at 18+ years of age or by parents and/or a responsible guardian of individuals under 18 years of age, was required for participation. Additionally, personal information, including name, age, sex, and address was noted. The clinical examination occurred in accordance with the guide for epidemiological surveys in oral health of the World Health Organization (WHO)[14], and in accordance with the Oral Health Brazil Project 2010 (manual) [15], by a single evaluator (C.V.R.M.) previously trained and calibrated in the pilot exercise with 20 repeated exams on subsequent days. The research excluded adolescents and young adults using anticonvulsant medications, immunosuppressants, or calcium channel blockers due to the proven influence of these drugs on periodontal tissue. Besides, female participants could not be pregnant.

### Variables, data sources, and measurement

The clinical variables considered in the samples were the presence (i) of periodontal disease and (ii) dental caries. The DMFT index was used to evaluate dental caries in all erupted teeth. Periodontal health conditions were evaluated by using the Community Periodontal Index (CPI) [15]. This assigns the following teeth as index—16, 11, 26, 36, 31, and 46 –in order to characterize the site condition. The Oral Health Impact Profile (OHIP-14) questionnaire, already validated in riverine rural populations [16], was the instrument used for measuring the subjective impacts of oral health on the quality of life. The OHIP-14 was individually completed after I provided directions to the participating group. It encompasses seven dimensions: functional limitation, physical pain, psychological discomfort, physical disability, psychological disability, social disability, and handicap. The survey contains 14 questions, and each allows results ranging from 0 to 4 points (0 = never, 1 = rarely, 2 = sometimes, 3 = repeatedly, 4 = always); the final value is obtained from the sum of 14 responses [16–19].

The mean total DMFT by sex and location was calculated, in addition to the mean number of decayed, missing, and filled teeth. For statistical analysis, the DMFT data were categorized as 0: DMFT = 0; 1: DMFT between 1 and 3; and 2: DMFT greater than 3. For the CPI, we considered separately the mean number of healthy sites, sites with bleeding and/or calculus, and the mean number of sites with shallow and deep pockets by sex and location.

For the OHIP outcome, we calculated the sum of the values attributed to each of the questions in the questionnaire, resulting in a discrete final variable for each individual. Thus, we obtained the mean and standard deviation by sex and for each location studied. Additionally, the prevalence of impact at each location was obtained by calculating the percentage of individuals who answered one or more question items with “fairly often” or “very often.” The OHIP value for each of its seven domains was also analyzed separately.

### Statistical methods

The replicability of the tests was analyzed using Kappa statistics. The weighted Kappa test was performed for the DMFT index (categorized) and for periodontal pockets (ordinal data). The conventional Kappa test was performed for bleeding and calculus (nominal data).

Due to the recognition of the dependence between observations within the same group, the multilevel model with Poisson regression was used for data analysis. The total scores of the OHIP (discrete quantitative variable) were the outcome variable. The rationale of rates and respective 95% confidence intervals were calculated with this approach. The STATA 12.0 program (Texas, USA) was used. We included in the final multivariate regression model all the variables that both presented p <0.05 in the univariate model and were considered of greater plausibility. For all analyses, we adopted a 5% level of significance.

## Results

The analysis of the replicability of the exams indicated a Kappa value of 0.91 (95CI = 0.76–1) for the DMFT index, 0.66 (95CI = 0.37–1) for gingival bleeding, 0.78 (95CI = 0.49–1) for the presence of calculus, and 0.65 (95IC = 0.57–1) for periodontal pockets.

### Participants

The sample obtained in the closest riverine community (8.3 km), Maracapucu, included all individuals within the determined age interval, except for 15 students (7%) who missed class on the data gathering dates and 11 students (5.2%) who refused to participate in the survey. In the Tucumanduba riverine community, located further away from town (28.3 km), nine students (5%) were absent when data was gathered, and six (3.3%) parents refused to sign the consent form. A proportional sample was obtained in the public school located in the urban area of Abaetetuba. The sample involved the students who were in school and agreed to participate in the exam. In addition, students answered a questionnaire to confirm that they had lived in the city for at least five years, which is a participation criterium for the urban sample due to the significant migration from the riverside to the urban area.

In the urban school, 107 boys (50.5%) and 105 girls (49.5%) were examined (Table 1). The highest mean within the DMFT index was observed for decayed teeth at 1.25 (SD 1.75) (Table 2). Female participants presented the highest mean for this condition at 1.40 (SD 1.90). Regarding the periodontal condition, the mean number of sites with bleeding and/or calculus of 3.05 (SD 1.61) was higher when compared to the mean of healthy sites, 2.70 (SD 1.62), and sites with periodontal pockets, 0.40 (SD 0.94).

**Table 1:** Sample populations: Number of students and relative frequency by sex and location.

	Urban school		Maracapucu riverine school		Tucumanduba riverine school		TOTAL	
	Male	Female	Male	Female	Male	Female	Male	Female
*n*	107	105	81	105	78	88	266	298
%	50,5%	49,5%	43,6%	56,4%	47,0%	53,0%	47,2%	52,8%
**Total**	**212**		**186**		**166**		**564**	

**Table 2:** Descriptive statistics for age, OHIP, DMFT, and periodontal condition by sex and location.

VARIABLES		LOCALITIES					
		Urban		Maracapucu Riverine		Tucumanduba Riverine	
		Mean (SD)		Mean (SD)		Mean (SD)	
SEX		Male	Female	Male	Female	Male	Female
AGE		16.93 (1.93)	16.62 (1.15)	16.81 (2.10)	16.50 (2.11)	17.15 (2.03)	16.78 (1.93)
		**16.78 (1.59)**		**16.64 (2.11)**		**16.96 (1.98)**	
OHIP	Functional limitation	0.67 (1.06)	0.54 (0.92)	0.86 (1.38)	0.76 (1.26)	0.72 (0.98)	0.65 (1.17)
		**0.61 (0.99)**		**0.81 (1.31)**		**0.68 (1.08)**	
	Physical pain	1.61 (1.46)	1.86 (1.68)	1.62 (1.76)	1.91 (1.78)	1.36 (1.33)	2.07 (1.92)
		**1.73 (1.57)**		**1.78 (1.77)**		**1.73 (1.70)**	
	Psychological discomfort	1.42 (1.97)	1.70 (2.24)	1.65 (2.26)	2.03 (2.36)	1.59 (2.00)	2.26 (2.42)
		**1.56 (2.11)**		**1.87 (2.32)**		**1.95 (2.25)**	
	Physical disability	0.65 (1.37)	0.69 (1.35)	0.89 (1.54)	0.93 (1.21)	0.64 (1.09)	0.82 (1.40)
		**0.67 (1.36)**		**0.91 (1.36)**		**0.73 (1.27)**	
	Psychological disability	0.78 (1.35)	0.87 (1.44)	1.39 (1.71)	1.46 (1.68)	1.08 (1.73)	1.27 (1.72)
		**0.82 (1.39)**		**1.43 (1.68)**		**1.18 (1.72)**	
	Social disability	0.43 (0.96)	0.58 (1.31)	1.02 (1.57)	1.09 (1.66)	0.82 (1.66)	1.11 (1.43)
		**0.50 (1.15)**		**1.06 (1.62)**		**0.98 (1.54)**	
	Handicap	0.34 (0.83)	0.40 (0.96)	1.12 (1.61)	0.89 (1.27)	0.69 (1.43)	0.98 (1.42)
		**0.37 (0.90)**		**0.99 (1.43)**		**0.84 (1.43)**	
**TOTAL OHIP**		5.90 (6.36)	6.65 (7.25)	8.57 (8.83)	9.07 (7.20)	6.91 (7.16)	9.16 (8.78)
		**6.27 (6.81)**		**8.85 (7.93)**		**8.10 (8.12)**	
DMFT	Decayed	1.10 (1.60)	1.40 (1.90)	3.33 (3.07)	2.57 (2.45)	1.67 (1.78)	1.60 (1.85)
		**1.25 (1.75)**		**2.90 (2.75)**		**1.63 (1.81)**	
	Missing	0.34 (0.78)	0.31 (0.64)	0.46 (0.95)	0.50 (0.79)	0.32 (0.89)	0.32 (0.75)
		**0.32 (0.71)**		**0.48 (0.86)**		**0.32 (0.82)**	
	Filled	0.45 (1.03)	0.55 (1.17)	0.17 (0.67)	0.32 (0.98)	0.10 (0.66)	0.19 (0.76)
		**0.50 (1.10)**		**0.26 (0.86)**		**0.15 (0.71)**	
**TOTAL DMFT**		1.89 (2.16)	2.27 (2.26)	3.96 (3.39)	3.40 (2.78)	2.09 (2.29)	2.11 (2.18)
		**2.07 (2.21)**		**3.64 (3.07)**		**2.10 (2.22)**	
PERIODONTAL CONDITION	Healthy subjects	2.86 (1.73)	2.54 (1.50)	1.17 (1.23)	1.66 (1.52)	2.36 (1.80)	2.44 (1.65)
		**2.70 (1.62)**		**1.45 (1.42)**		**2.40 (1.72)**	
	Bleeding/calculus subjects	2.87 (1.72)	3.23 (1.48)	4.06 (1.37)	3.71 (1.56)	3.19 (1.85)	3.07 (1.75)
		**3.05 (1.61)**		**3.87 (1.49)**		**3.13 (1.79)**	
	Subjects with pocket	0.49 (1.05)	0.31 (0.80)	1.46 (1.41)	1.15 (1.23)	0.19 (0.56)	0.18 (0.75)
		**0.40 (0.94)**		**1.28 (1.31)**		**0.19 (0.67)**	

OHIP- Functional limitation (1° dimension); Physical pain (2° dimension); Psychological discomfort (3° dimension); Physical disability (4° dimension); Psychological disability (5° dimension); Social disability (6° dimension); Handicap (7° dimension).

Bold numbers–group means (standard deviations).

At the Maracapucu riverine school, 81 boys (43.54%) and 105 girls (56.45%) were examined. In the Tucumanduba riverine school, 78 boys (46.98%) and 88 girls (53.01%) were examined (Table 1). In relation to the DMFT variable, both riverine communities presented a predominance of decayed teeth, with an average of 2.90 (2.75) for Maracapucu and 1.63 (1.81) for Tucumanduba (Table 2). Considering the periodontal indicators, the higher averages were of sites affected by bleeding and calculus at 3.87 (1.49) for Maracapucu and 3.13 (1.79) for Tucumanduba.

### Outcome data

The OHIP presented higher values in the riverine communities, with an average of 8.85 (SD 7.93) in the community of Maracapucu and 8.10 (SD 8.12) in Tucumanduba. For the urban area, the OHIP average was 6.27 (SD 6.81) (Table 2). In all locations, the highest OHIP averages were concentrated in the female participants and in the domains that refer to mouth or tooth pain, concern with the oral overall condition itself, as well as concern in relation to the problem, the 2nd and 3rd OHIP domains. From the calculation of impact prevalence, the values were (i) 61.5% (54.0%–68.9%) for the most remote riverine community (Tucumanduba), (ii) 75.8% (69.7%–82.0%) for the nearest riverine community (Maracapucu), and (iii) 48.2% (41.4%–54.8%) for the urban location.

### Main results

Considering the univariate statistical analysis, all variables displayed a significant association with the OHIP outcome (Table 3). In the analysis of the multivariate model, the variables of gender and age presented a significant relation with the OHIP outcome (p <0.05), with the female sex being more significant (RR = 1.19, 95CI = 1.12–1.27). The association between the riverine school that was nearest to the city with the OHIP was also significant (Rate Ratio = 1.17, 95CI = 1.08–1.27), and the riverine school further away from the urban center presented an even higher rate ratio for the impact on oral health-related quality of life (RR = 1.31, 95CI = 1.21–1.42).

**Table 3:** Multilevel Poisson regression analysis for the association between explanatory variables and total OHIP scores.

Explanatory variables	Unadjusted rate ratio (95% CI)	*P*-value	Adjusted rate ratio (95%CI)	*P*-value
**1**° **level: Adolescents (*n* = 564)**				
**Sex**				
Male	1.00		1.00	
Female	1.16 (1.09–1,23)	<0.001	1.19 (1.12–1.27)	<0.001
**Age**	1.06 (1.05–1.08)	<0.001	1.04 (1.02–1.05)	<0.001
**DMFT**				
DMFT 0	1.00		1.00	
DMFT 1	1.26 (1.16–1.37)	<0.001	1.25 (1.15–1.36)	<0.001
DMFT 2	1.63 (1.50–1.77)	<0.001	1.53 (1.41–1.67)	<0.001
**Periodontal health**	1.00		1.00	
**Bleeding**	1.03 (1.01–1.05)	0.001	0.99 (0.96–1.02)	0.494
**Calculus**	1.03 (1.01–1.05)	0.001	1.02 (0.99–1.04)	0.160
**Shallow pocket**	1.08 (1.05–1.12)	<0.001	1.07 (1.03–1.10)	<0.001
**Deep pocket**	1.26 (1.18–1.34)	<0.001	1.15 (1.07–1.24)	<0.001
**2**° **level: School (*n* = 564 adolescents)**				
**School**				
Urban school	1.00		1.00	
Maracapucu riverine school	1.41 (1.31–1.52)	<0.001	1.17 (1.08–1.27)	<0.001
Tucumanduba riverine school	1.29 (1.20–1.39)	<0.001	1.31 (1.21–1.42)	<0.001

95% CI = 95% confidence interval

All variables were included in the adjusted model.

In regards to the DMFT index, participants with a DMFT between 1 and 3 had a higher impact on OHIP-14 than individuals without caries (RR = 1.25, 95CI = 1.15–1.36). There was an even more significant relationship between DMFT category 2 (DMFT> 3) and the OHIP (RR = 1.53, 95CI = 1.41–1.67). In relation to the periodontal indicators, the associations “shallow pocket x OHIP” (RR = 1.07, 95CI = 1.03–1.10) and “deep pocket x OHIP” (RR = 1.15, 95CI = 1.07–1.24) were significant, whereas the variables of bleeding (p = 0.494) and calculus (p = 0.160) were not significantly associated with the outcome (Table 3).

## Discussion

An impact prevalence of 61.5% (54.0%–68.9%) and OHIP mean of 8.10 (SD = 8.12) were obtained for the most remote riverine community. For the nearest one, the impact prevalence was 75.8% (69.7%–82.0%), with a mean OHIP of 8.85 (SD = 7.93). Moreover, for the urban area, the prevalence was 48.2% (41.4%–54.8%) and the OHIP mean was 6.27 (6.81). The obtained values are significantly higher when compared to studies performed in other Brazilian communities and developed countries [20–26].

In comparison, in a survey conducted with people aged 18 years and older from Australia and England, the impact prevalence of the oral condition on the quality of life was 18.2% (16.2%–20.2%) and 15.9% (14.4%–17.4%), respectively [23]. A study with adults from Southern Brazil recorded an impact prevalence for the worst OHIP score of approximately 16% [24], similar to that found in the study conducted in England. A survey applied to adolescents between 15 and 17 years of age living in the urban area of Santa Catarina, Brazil, recorded a mean OHIP of 3.95 (SD = 4.88) [21], which is a lower value compared to our finding in the remote communities of the Amazon (Table 2). An impact prevalence of 70.3%, similar to that found in this study, was observed in a study conducted with individuals from rural areas in the State of Pernambuco, Northeast Brazil [27].

### Interpretation

Findings in this study imply that oral problems have deep social and economic roots [20, 28, 29]. Furthermore, cultural and geographic factors are also highly associated with patterns of health conditions, as well as with subjective recognition of the disease. This can be specifically observed among isolated populations, with specific habits and cultures, such as rural or riverine communities.

In the Amazon region, several riverine communities do not have basic services that guarantee the right to local health care, causing people to travel to the nearest city. The financial cost of traveling is usually high for riverine individuals. Consequently, this cost is usually not paid until after the pathology is already in advanced stages, with the presence of intense pain. In addition, commuting depends on the river flow and tides, which refrain trips to a small part of the day and causes riverine individuals to be tardy for appointments at the nearest health center, considering the fact that appointments are based on first come, first serve. Unfortunately this fact was reported by many respondents as a frequent occurrence for all health related visits to urban centres.

Based on a literature review, the majority of studies that evaluate a population’s degree of satisfaction with its oral health status are carried out in urban centers and do not take into account specific conditions in communities that inhabit more isolated areas. When considering issues faced by the rural/riverine populations such as geographic isolation and the consequent distance from urban cultural and social patterns, general health conditions such as nutritional and sanitation deficiencies, and the distance from social networks and media, one could conclude that these populations would report a lower impact of their oral health status on quality of life. However, the impact prevalence indicated that individuals in the Amazon riverine communities examined in the present study complained of a greater impact of oral health on the quality of life when compared to individuals in the urban community.

It is likely that the greatest impact on oral health-related quality of life found among riverine communities occurred due to (i) the absence of health care within the community, (ii) the distance from these communities to the city, and (iii) limitations in transportation systems. These elements, which generate demand for dental care and increase the risk of pain from dental origin, are the most frequently reported in the OHIP questionnaire in association with worse quality of life.

The largest association of the OHIP with female participants is in accordance with other studies in which females are more attentive to their oral health conditions and more sensitive to negative impacts than males. Other studies also show that women are more likely to seek care than men [7], are more dissatisfied with their appearance related to their oral condition [30], and perceive more damages related to oral tissues [31,32]. Age has been associated with the quality of life by OHIP-14, although with lower impact (RR = 1.04, 95CI = 1.02–1.05). Older individuals tend to better recognize or understand their own health condition. Other studies indicate that older individuals report less impact when considering aesthetic factors and dental sensitivity, while younger individuals are more anxious about their dental condition [7].

The highest mean DMFT scores were observed for individuals in the riverine communities: 3.64 for Maracapucu and 2.10 for the community living along the Tucumanduba River. A study conducted in 1990 among riverine children from the Tucumanduba River from 7 to 14 years of age reported a mean DMFT of 6.5, which is considered quite high when compared to WHO parameters [33, 34]. These data reveal a drastic reduction in caries rates in these communities in the last decades.

The reduction of dental caries in Brazil occurred throughout the country. In 2003, Brazil had a DMFT index of 2.8, changing to 2.1 in 2010. For the 15–19 age group, the DMFT fell even more, from 6.1 in 2003 to 4.2 in 2010. According to the Oral Health Brazil Project 2010 –National Oral Health Survey [3], the country is in the group of low caries index countries, where the DMFT indicator should be between 1.2 and 2.6, according to the WHO classification.

The periodontal conditions of bleeding and calculus were not significantly associated with the OHIP. This is probably due to the multicollinearity of the periodontal variables and to the cumulative effect of the periodontal pocket condition, which implies a previous bleeding condition and calculus accumulation. These conditions represent initial periodontal changes, which are avoidable by improvement in oral hygiene, and indicate a lack of preventive activities in oral health.

The presence of a shallow or deep periodontal pocket—a condition that represents more advanced stages of disease and indicates absence of treatment—was higher among the Maracapucu riverine community, with a mean of 1.28 sites affected, followed by the urban location (mean of 0.40) and, finally, by Tucumanduba, the more remote community, with a mean of 0.19 sites with periodontal pockets. The periodontal data obtained in Tucumanduba, along with the considerable number of individuals enrolled in the DMFT 0 score, are probably due to the preventive actions of volunteer dentists in the area at least once a year, a fact not reported in the Maracapucu community.

### Limitations

The most remote riverine location presented a greater number of individuals with higher sums of OHIP scores. This greater impact may also be due to some other variable not examined in the research, such as dental malocclusion. Alternatively, the accumulation of many other basic issues in these communities can overlap with some dental needs. This fact can be present in both riverine communities since they share similar life conditions, and can be considered a limiting factor of this study.

The moderate Kappa value that was obtained should also be considered a limitation of the study. This can be partially explained by limited lighting in the clinical exam and for the absence of supporting equipment.

### Generalizability

According to the adjusted regression model, the riverine community of Tucumanduba, which is further away from town, demonstrated a greater association with poor quality of life. This suggests that geographic distance and consequent difficulty in accessing health services may generate interferences in the oral health-related quality of life. A possible reason is the prolonged time without treatment. In addition, the presence of periodontal pockets and a DMFT greater than 3 presented a more significant relationship with the OHIP. It seems reasonable to extrapolate this finding to other remote communities, so it is assumed that the more advanced the oral pathology and the longer the time elapsed without care, the greater the reported impact.

Our findings revealed that dental caries and periodontal disease have a major impact on the quality of life. Riverine communities, which are further away from urban centers, report a greater impact of the oral condition on the quality of life, probably due to the difficulty in obtaining care and consequent prolongation of episodes of pain.

Timely dental care reduces the duration of illness or the discomfort and disability related to it. Improved access to health services and the development of prevention programs in basic health care, targeted at the real needs of isolated and economically disadvantaged populations, could reduce health inequities.

## Supporting information

S1 File Approval of the Research Ethics Committee of the Health Sciences Institute of the Federal University of Pará. (PDF)

## References

[1] Cohen-Carneiro F, Souza-Santos R, Pontes DG, Salino AV, Rebelo MAB. Supply and use of oral health services in Amazonas, Brazil: a case study in a riverine population in the municipality of Coari. Cad. Public Health. 2009;25:1827–1838. 10.1590/s0102-311x2009000800019 19649424

[2] Silva RHA, Castro RFM, Cunha DCS, Almeida CT, Bastos JRM, Camargo LMA. Dental caries in a riverine population of the State of Rondônia, Amazon Region, Brazil, 2005/2006. Cad. Public Health. 2008;24(10):2347–2353. 10.1590/s0102-311x2008001000015 18949236

[3] Brazil. Ministry of Health. Secretariat of Health Care. Department of Basic Attention. SB Brazil Project 2010: National Oral Health Survey—Main Results. Brasília, DF: Ministry of Health; 2011.

[4] Borges CM, Campos ACV, Vargas AMD, Ferreira EF. Profile of dental losses in adults according to social capital, demographic and socioeconomic characteristics. Ciênc. Collective Health. 2014;19(6):1849–1858. 10.1590/1413-81232014196.02332013 24897484

[5] Mota JC, Valente JG, Schramm JMA, Leite IC. Study of the disease burden of oral conditions in Minas Gerais, Brazil, 2004–2006. Ciênc. Collective Health. 2014;9(7):2167–2178. 10.1590/1413-81232014197.09312013 25014296

[6] Bianco VC, Lopes ES, Borgato MH, Silva PM, Marta SN. The impact of oral conditions on the quality of life of people with fifty or more years of life. Ciênc. Collective Health. 2010;15(4):2165–2272. 10.1590/s1413-81232010000400030 20694338

[7] Cohen-Carneiro F, Souza-Santos R, Rebelo MAB. Quality of life related to oral health: contribution of social factors. Ciênc. Collective Health. 2011;16(Suppl.1):1007–1015. 10.1590/s1413-81232011000700033 21503449

[8] Lion A, Sheiham A. Relationship between dental clinical status and subjective impacts on daily life. J. Dent. Res. 1995;74:1408–1413. 10.1177/00220345950740071301 7560392 10.1177/00220345950740071301

[9] Ibge. An overview of health in Brazil. Access and use of services, health conditions and risk factors and health protection 2008. PNAD 2008. Rio de Janeiro: IBGE; 2010. 245 p.

[10] Singh A, Harford J, Antunes JLF, Peres MA (2018) Area-level income inequality and oral health among Australian adults-A population- based multilevel study. PLoS ONE 13(1): e0191438 10.1371/journal.pone.0191438. 29364943 PMC5783384

[11] Bhandari B, Newton JT, Bernabe E. Income Inequality and Use of Dental Services in 66 Countries. J Dent Res. 2015; 94(8):1048–54. Epub 2015/05/23. 10.1177/0022034515586960 . 25994178

[12] Von Elm E, Altman DG, Egger M, Pocock SJ, Gøtzsche PC, Vandenbroucke JP; STROBE Initiative. The strengthening the reporting of observational studies in epidemiology (STROBE) statement: guidelines for reporting observational studies. J Clin Epidemiol. 2008;61(4):344–349. 10.1016/j.jclinepi.2007.11.008 18313558 10.1016/j.jclinepi.2007.11.008

[13] Ibge. Research Directorate. Coordination of Population and Social Indicators. Available from: http://www.ibge.gov.br/home/estatistica/populacao/estimativa2016/estimativa_tcu.shtm. Accessed November 1, 2016.

[14] World Health Organization. Oral Health Surveys–basic methods- 5th ed World Health Organization; 2013.

[15] Brazil. Ministry of Health. Secretariat of Health Care. Department of Basic Attention. SB Brazil Project 2010: National Oral Health Survey—field team manual. Brasília, DF: Ministry of Health; 2009.

[16] Cohen-Carneiro F, Rebelo MAB, Souza-Santos R, Ambrosano GMB, Salino AV, Pontes DG. Psychometric properties of OHIP-14, prevalence, and severity of impacts on oral health in a rural riverine population in the state of Amazonas, Brazil. Cad. Saúde Pública, Rio de Janeiro. 2010;26(6):1122–1130. 10.1590/s0102-311x2010000600006 20657977

[17] Gabardo MCL, Moysés ST, Moysés S. Oral health self-perception according to the Oral Health Impact Profile (OHIP) and associated genera: systematic review. Rev. Panam. Public Health. 2013;33(6):439–445. 23939370

[18] Slade GD. Derivation and validation of a short oral health impact profile. Community Dent. Oral Epidemiol. 1997;25:284–290. 9332805 10.1111/j.1600-0528.1997.tb00941.x

[19] Oliveira BH, Nadanovsky P. Psychometric properties of the Brazilian version of the Oral Health Impact Profile—Short form. Community Dent. Oral Epidemiol. 2005;33:307–314. 10.1111/j.1600-0528.2005.00225.x 16008638

[20] Miotto MHMDB Barcellos LA, Velten DB. Evaluation of the impact on quality of life caused by oral problems in the adult and elderly population in the municipality of the Southeast region. Ciênc. Collective Health. 2012;17(2):397–406. 10.1590/s1413-81232012000200014 22267035

[21] Biazevic MGH, Rissoto RR, Crosato EM, Mendes LA, Mendes MOA. Relationship between oral health and its impact on quality of life among adolescents. Braz. Oral Res. 2008;22(1):36–42. 18425243 10.1590/s1806-83242008000100007

[22] Guerra MJC, Greco RM, Leite ICG, Ferreira EF, De Paula MVQ. Impact of oral health conditions on workers' quality of life. Ciênc. Collective Health. 2014;19(12):4777–4786. 10.1590/1413-812320141912.21352013 25388186

[23] Slade GD, Nuttall N, Sanders AE, Steele JG, Allen PF, Lahti S. Impacts of oral disorders in the United Kingdom and Australia. Br. Dent. J. 2005;198:489–493. 10.1038/sj.bdj.4812252 15849587 10.1038/sj.bdj.4812252

[24] Gabardo MCL, Moysés SJ, Moysés ST, Olandoski M, Olinto MTA, Pattussi MP. Multilevel analysis of self-perception in oral health and associated factors in Southern Brazilian adults: a cross-sectional study. Cad. Saúde Pública, Rio de Janeiro 2015;31(1):49–59. 10.1590/0102-311x00037814 25715291

[25] Sheiham A, Steele JG, Marcenes W, Tsakos G, Finch S, Walls AWG. Prevalence of impacts of dental and oral disorders and their effects on eating among older people; a national survey in Great Britain. Community Dent. Oral Epidemiol. 2001;29:195–203. 11409678 10.1034/j.1600-0528.2001.290305.x

[26] Leão MM, Garbin CAS, Moimaz SAS, Rovida TAS. Oral health and quality of life: an epidemiological survey of adolescents from settlement in Pontal do Paranapanema/SP, Brazil. Ciênc. Collective Health. 2015;20(11):3365–3374. 10.1590/1413-812320152011.00632015 26602714

[27] Santillo PMH, Gusmão ES, Moura C, Soares RSC, Cimões R. Factors associated with dental loss among adults in rural areas of the state of Pernambuco, Brazil. Ciênc. Collective Health. 2014;19 (2):581–590. 10.1590/1413-81232014192.20752012 24863834

[28] Sheiham A, Tsakos G. Assessing the needs of the socio-odontological approach In: Pinto VG, editor. Collective Oral Health. 5th ed São Paulo: Santos; 2008 287–316.

[29] Silva RHA, Castro RFM, Caldana ML, Sales-Peres A, Sales-Peres SHDC, Bastos JRM. Challenges in oral health promotion: anthropological-cultural and epidemiological approach of riverine population in RO. Braz. Oral. Res. 2004;18(Suppl):29.15273783

[30] Klages U, Bruckner A, Zentner A. Dental aesthetics, self-awareness, and oral health-related quality of life in young adults. Eur. J. Orthod. 2004;26:507–514. 15536839 10.1093/ejo/26.5.507

[31] Chavers LS, Gilbert GH, Shelton BJ. Racial and socioeconomic disparities in oral health, a measure of oral health-related quality of life: 24-month incidence. J. Public Health Dent. 2002;62:140–147. 12180041 10.1111/j.1752-7325.2002.tb03435.x

[32] John MT, Koepsell TD, Hujoel P, Miglioretti DL, Leresche L, Micheelis W. Demographic factors, dental status and oral health-related quality of life. Community Dent. Oral Epidemiol. 2004;32:125–132. 10.1111/j.0301-5661.2004.00144.x 15061861 10.1111/j.0301-5661.2004.00144.x

[33] Normando ADC, Araújo ICde. Prevalence of dental caries in a population of schoolchildren from the Amazon region. Rev. Saúde Pública, S. Paulo 1990;24:294–299. 10.1590/s0034-89101990000400007 2103647

[34] Ibge. National Health Survey 2013: Access and use of health services, accidents and violence. Brazil, Major Regions and Federation Units. Rio de Janeiro: IBGE; 2015.

