# Supplementary material for: The impact of oral health on quality of life of urban and riverine populations of the Amazon: A multilevel analysis
Source: PLoS One. 2018 Nov 30;13(11):e0208096. doi: 10.1371/journal.pone.0208096 (PMC6267970; doi:10.1371/journal.pone.0208096)
Supplement: S1 File — (PDF) [file pone.0208096.s001.pdf]

PARECER CONSUBSTANCIADO DO CEP

DADOS DO PROJETO DE PESQUISA

Título da Pesquisa: CONDIÇÃO DE SAÚDE BUCAL E SEUS IMPACTOS NA QUALIDADE DE VIDA: ANÁLISE COMPARATIVA ENTRE ADOLESCENTES DE POPULAÇÃO URBANA E RIBEIRINHA DE MUNICÍPIO DA AMAZÔNIA.

Pesquisador: CAMILA DE VASCONCELLOS ROCHA MAIA

Área Temática:

Versão: 1

CAAE: 56863116.4.0000.0018

Instituição Proponente: Instituto de Ciências da Saúde da Universidade Federal do Pará - ICS/ UFPA

Patrocinador Principal: Financiamento Próprio

DADOS DO PARECER

Número do Parecer: 1.593.113

Apresentação do Projeto:

Apesar de reconhecido e garantido o acesso à saúde como direito de todos e dever do Estado, as desigualdades regionais e as carências de cobertura dos serviços de saúde são ainda uma realidade no país. No que diz respeito à saúde bucal, grande parcela da população brasileira ainda nos dias de hoje não tem acesso a atendimentos odontológicos. O último levantamento epidemiológico de saúde bucal no Brasil evidenciou para a

Região Norte uma maior proporção de indivíduos que nunca foram ao dentista, maior intervalo de tempo desde a última consulta e maior proporção de consultas motivadas pela dor do que as regiões Sul e Sudeste do país. A Cárie Dentária e a Doença Periodontal estão entre as doenças de maior ocorrência na cavidade bucal e, portanto, entre as de maior interesse da saúde coletiva. Fatores socioeconômicos e geográficos apresentam

influência nas oportunidades de acesso aos serviços de saúde, sendo assim, variações importantes nas características de utilização dos serviços entre populações mais isoladas, como as rurais/ribeirinhas, e urbanas são muito prováveis. Diferenças nos níveis de informação e esclarecimentos em saúde, formas de reconhecimento subjetivo do processo saúde-doença e impactos na qualidade de vida dessas populações são outros aspectos que em análises qualitativas, podem ser esperados. Indivíduos de populações ribeirinhas apresentam dificuldades

de acesso às cidades em virtude do isolamento geográfico e dificuldades de transporte, e apresentam alimentação basicamente dependente da pesca e pequena agricultura de subsistência. Este trabalho busca diagnosticar e quantificar a prevalência de Doença Periodontal e Cárie dentária por meio dos índices CPI e CPOD,em indivíduos na faixa etária de 15 a 25 anos, matriculados na rede pública de ensino e em programas de educação para jovens e adultos,residentes em área urbana há pelo menos 5 anos e rural/ribeirinha,do município de Abaetetuba – PA, município pólo da Região do Baixo Tocantins,com grande número de ilhas habitadas. Serão analisados os impactos das condições de saúde bucal na qualidade de vida destes indivíduos por meio da aplicação do instrumento OHIP-14 em sua versão simplificada e avaliados os resultados de forma comparativa entre as populações,verificando a existência ou não de diferenças relevantes no que se refere à prevalência das doenças consideradas e aos impactos na qualidade de
vida entre estas populações.

Objetivo da Pesquisa:

Objetivo Primário:

Diagnosticar e quantificar a prevalência de Doença Periodontal e Cárie Dentária por meio dos índices CPI, CPO-D, em indivíduos na faixa etária de 15 a 25 anos de idade, vinculados à rede pública de ensino, residentes em área urbana e rural/ribeirinha do município de Abaetetuba-PA. Analisar os impactos da condição de saúde bucal na qualidade de vida destas populações por meio da aplicação do OHIP-14 em análise comparativa entre
os residentes de área urbana e os habitantes de regiões ribeirinhas.

Avaliação dos Riscos e Benefícios:

Riscos:

Os riscos da pesquisa são considerados mínimos. Em relação ao risco físico, o exame realizado não causará nenhuma dor nem risco de biossegurança. Os riscos psicológicos também são mínimos. Para minimizá-los os participantes da pesquisa terão a garantia de que as informações obtidas serão analisadas com a preocupação de evitar a identificação dos mesmos e que eles terão a liberdade de retirar seu consentimento a qualquer momento e deixar de participar do estudo, sem qualquer prejuízo.

Benefícios:

Definir as necessidades de tratamento de uma população através de estudos epidemiológicos, análise de determinantes sociais e compreensão mais subjetiva dos impactos causados pelas condições de saúde bucal na qualidade de vida, são importantes passos para o planejamento e execução de políticas e programas de saúde. Esses estudos, amplamente realizados em

populações de centros urbanos, ainda são pouco aplicados
em populações específicas mais isoladas como comunidades ribeirinhas. Além disso,a participação dos envolvidos trará como benefício aos mesmos, o conhecimento de sua condição bucal e em caso de existência de patologias bucais, ele será referenciado a Referências Secundárias e terciárias de Saúde Bucal do Sistema Único de Saúde para tratamento e acompanhamento. O estudo também proporcionará um melhor conhecimento sobre a cárie dentária e a doença periodontal na região estudada.

Comentários e Considerações sobre a Pesquisa:

O protocolo apresentado dispõe de metodologia e critérios definidos conforme resolução 466/12 do CNS/MS.

Considerações sobre os Termos de apresentação obrigatória:

Os termos apresentados contemplam os sugeridos pelo sistema CEP/CONEP.

Conclusões ou Pendências e Lista de Inadequações:

Diante do exposto somos pela aprovação do protocolo. Este é nosso parecer, SMJ.

Considerações Finais a critério do CEP:

Este parecer foi elaborado baseado nos documentos abaixo relacionados:

Tipo Documento	Arquivo	Postagem	Autor	Situação
Informações Básicas do Projeto	PB_INFORMAÇÕES_BÁSICAS_DO_PROJETO_569328.pdf	24/05/2016 20:18:18		Aceito
Outros	007.jpg	24/05/2016 20:17:06	CAMILA DE VASCONCELLOS ROCHA MAIA	Aceito
Projeto Detalhado / Brochura Investigador	Projeto_PPGSAS.doc	16/05/2016 10:42:04	CAMILA DE VASCONCELLOS ROCHA MAIA	Aceito
Outros	003.jpg	01/02/2016 12:33:53	CAMILA DE VASCONCELLOS ROCHA MAIA	Aceito
Outros	002.jpg	01/02/2016 12:32:27	CAMILA DE VASCONCELLOS ROCHA MAIA	Aceito
Outros	001.jpg	01/02/2016 12:26:04	CAMILA DE VASCONCELLOS ROCHA MAIA	Aceito
TCLE / Termos de Assentimento /	TCLE.docx	08/01/2016 12:21:55	CAMILA DE VASCONCELLOS	Aceito

Justificativa de Ausência	TCLE.docx	08/01/2016 12:21:55	ROCHA MAIA	Aceito
Declaração de Instituição e Infraestrutura	declaracaoescolaribeirinha2.pdf	14/11/2015 22:27:27	CAMILA DE VASCONCELLOS ROCHA MAIA	Aceito
Declaração de Instituição e Infraestrutura	declaracaoescolaribeirinha.pdf	14/11/2015 22:24:26	CAMILA DE VASCONCELLOS ROCHA MAIA	Aceito
Declaração de Instituição e Infraestrutura	declaracaoescolaurbana.pdf	14/11/2015 22:22:33	CAMILA DE VASCONCELLOS ROCHA MAIA	Aceito
Folha de Rosto	folhaderostoplataforma.pdf	14/11/2015 21:38:08	CAMILA DE VASCONCELLOS ROCHA MAIA	Aceito

Situação do Parecer:
Aprovado

Necessita Apreciação da CONEP:
Não

BELEM, 10 de Junho de 2016

Assinado por:
Wallace Raimundo Araujo dos Santos
(Coordenador)

Endereço: Rua Augusto Corrêa nº 01-Sl do ICS 13 - 2º and.
Bairro: Campus Universitário do Guamá **CEP:** 66.075-110
UF: PA **Município:** BELEM
Telefone: (91)3201-7735 **Fax:** (91)3201-8028 **E-mail:** cepccs@ufpa.br
